# Association between chronic pain and risk of incident dementia: findings from a prospective cohort

**DOI:** 10.1186/s12916-023-02875-x

**Published:** 2023-05-04

**Authors:** Jing Tian, Graeme Jones, Xin Lin, Yuan Zhou, Anna King, James Vickers, Feng Pan

**Affiliations:** 1 Menzies Institute for Medical Research, University of Tasmania, Private Bag 23, TAS Hobart, 7000 Australia; 2 Wicking Dementia Research and Education Centre, University of Tasmania, Private Bag 23, TAS Hobart, 7000 Australia

**Keywords:** Chronic pain, Musculoskeletal pain, Multisite pain, Incidence, Dementia

## Abstract

**Background:**

Chronic musculoskeletal pain has been linked to dementia; however, chronic pain typically occurs in multiple sites; therefore, this study was to investigate whether greater number of chronic pain sites is associated with a higher risk of dementia and its subtypes.

**Methods:**

Participants (*N* = 356,383) in the UK Biobank who were dementia-free at baseline were included. Pain in the hip, knee, back, and neck/shoulder or ‘all over the body’ and its duration were assessed. Participants were categorised into six groups: no chronic pain; chronic pain in 1, 2, 3, and 4 sites, and ‘all over the body’. All-cause dementia and its subtypes were ascertained using hospital inpatient and death registry records. Cox regression was used to investigate the associations between the number of chronic pain sites and the incidence of all-cause dementia and its subtypes.

**Results:**

Over a median follow-up of 13 years, 4959 participants developed dementia. After adjustment for sociodemographic, lifestyle, comorbidities, pain medications, psychological problems, and sleep factors, greater number of chronic pain sites was associated with an increased risk of incident all-cause dementia (hazard ratio [HR] = 1.08 per 1 site increase, 95% CI 1.05–1.11) and Alzheimer’s disease (AD) (HR = 1.09 per 1-site increase, 95% CI 1.04–1.13) in a dose–response manner but not vascular and frontotemporal dementia. No significant association was found between the number of chronic pain sites and the risk of incident all-cause dementia among a subsample that underwent a fluid intelligence test.

**Conclusions:**

Greater number of chronic pain sites was associated with an increased risk of incident all-cause dementia and AD, suggesting that chronic pain in multiple sites may contribute to individuals’ dementia risk and is an underestimated risk factor for dementia.

**Supplementary Information:**

The online version contains supplementary material available at 10.1186/s12916-023-02875-x.

## Background

Dementia, characterised by progressive cognitive decline, is a major public health concern, with substantial impacts on the person with dementia as well as society, the economy, and healthcare systems [1]. Alzheimer’s disease (AD) is the major disease leading to dementia. Currently, there is no cure or effective disease-modifying treatment available. Consequently, identifying and targeting modifiable risk factors for dementia to implement preventive strategies are urgent needs [2].

Musculoskeletal pain is common in the general population, leading to limited physical function, poor quality of life, and disability [3]. It is a major public health concern, with an estimated prevalence of 74% in community-dwelling older adults [4–6]. Musculoskeletal pain often occurs in multiple sites, with studies including our own reporting 41–75% of people with musculoskeletal pain experiencing pain in two or more sites [6–9]. Compared to single-site pain, pain in multiple sites was associated with poorer physical and mental health [10, 11] as well as worse health-related outcomes [12–14].

Few cohort studies investigated the associations of musculoskeletal pain with cognitive decline and dementia, with some showing that pain is related to memory decline, accelerated cognitive decline [15, 16], or increased risk of dementia [17–20]. Although the precise mechanisms are not yet clear, several mechanisms by which pain contributes to cognitive impairment or dementia have been proposed. For example, it has been hypothesised that pain-induced neuroinflammation may be implicated in AD [21], and pain may divert attention by directly competing for cognitive processing resources [22]. In addition, pain may contribute to structural and functional changes in the brain which are also involved in cognitive function [23–25], although pain processing itself has a cognitive-evaluative component, and thus, they may share an inherent overlap. Living with chronic pain may also undermine the resilience of the brain to the accumulating burden of dementia-related pathology on the brain [26].

In contrast, others have reported no association between pain and cognitive impairment or dementia [27, 28] and suggested that pain may be a correlate or prodromal symptom rather than a cause of dementia [29]. The potential reasons for the inconsistency between studies may be due to variations in the study design, study population, follow-up length, and pain and outcome measures. For instance, most prior studies had a short (≤ 5 years) follow-up period among individuals aged ≥ 65 years [17–19, 27], did not assess the chronicity of pain [17–20, 27], and used different criteria to define dementia cases. Other than these, differences in controlling for confounding factors in previous studies could be one of the possible explanations, for example, no or few studies have considered some important factors such as sleep problems [30] and opioid use [31]. Thus, it remains unclear whether the excess risk is attributable to these potential confounding factors.

Currently, there is no study exploring the associations between chronic pain in multiple sites and risk of incident dementia as well as its subtypes. Therefore, this study examined whether pain in multiple sites increased the risk of all-cause dementia, AD, vascular, and frontotemporal dementia using large-scale data from a population-based cohort study where participants were followed for a period of 13 years with chronic pain assessed using a standardised definition, dementia cases and its subtypes ascertained by validated methods, and a wide range of potential confounders measured. Since chronic pain has been linked to cognitive impairment and dementia, we, therefore, hypothesised that chronic pain in multiple sites is associated with an increased risk of dementia and its subtypes in a dose–response relationship.

## Methods

### Study population

This study utilised data from the UK Biobank study, which is a large, population-based prospective cohort, with > 500,000 participants (aged 40–69 years) who attended one of 22 assessment centres across the UK between 2006 and 2010. A detailed description of the UK Biobank study was previously published [32]. The UK Biobank study was approved by the North West Multi-centre Research Ethics Committee, and all participants provided electronic informed consent. The current analyses were restricted to participants without self-reported or prevalent dementia at baseline.

### Pain assessment (exposure)

Participants were asked at baseline whether they had experienced pain at the sites of the hip, knee, back, and neck/shoulder in the last month that interfered with their usual activities. The question was asked via a touchscreen pain questionnaire, where more than one site could be selected. Participants could also choose pain ‘all over the body’, but they could not choose self-selected individual pain sites. If participants reported pain in at least one site or pain ‘all over the body’, they were then asked whether the reported pain had lasted for ≥ 3 months. Based on these questions, the number of painful sites which lasted for ≥ 3 months was summed to create a total number of chronic pain sites which categorised participants into six groups: no chronic pain; 1, 2, 3, and 4 sites of chronic pain; and chronic pain ‘all over the body’.

### Dementia diagnosis (outcomes)

All-cause dementia and its subtypes were ascertained using hospital inpatient and death registry records. Hospital inpatient records contained data on admissions and diagnoses obtained from Hospital Episode Statistics for England, Scottish Morbidity Record for Scotland, and Patient Episode Database for Wales. Death registry records were obtained from National Health Service (NHS) Digital for England and Wales and Information and Statistics Division for Scotland also provided additional cases. The International Classification of Diseases (ICD9 and ICD10) coding system was used to record primary and secondary hospital diagnoses and causes of death according to a set of algorithms developed by the UK Biobank outcome adjudication group (Additional file 1: Table S1). The algorithmically defined all-cause dementia and its subtypes (including AD, vascular dementia, and frontotemporal dementia) as outcomes for the current analyses were validated by the UK Biobank outcome adjudication group [33].

### Covariates

A range of baseline variables which are associated with both chronic pain and dementia were considered potential confounding factors based on existing literature within the field [1, 34], including (1) sociodemographic factors—age, sex, ethnicity (White and non-White), highest education qualification, and household income; (2) body mass index (BMI) and lifestyle factors—smoking status, frequency of alcohol consumption, and recommended moderate/vigorous physical activity (PA) (yes/no); (3) health factors—presence of any comorbidities (including hypertension, diabetes, stroke, cardiovascular disease, cancer, and lung disease), use of non-steroidal anti-inflammatory drugs (NSAIDs) (Additional file 1: Supplementary Text 1), and use of opioid (Additional file 1: Supplementary Text 2); and (4) psychological problems and sleep duration (Additional file 1: Supplementary Text 3). The presumed relationships between the selected covariates and pain/dementia are shown in Additional file 1: Fig. S1. Covariate subsets were selected and adjusted in the models based on their presumed relationships to avoid misinterpretation of the effects of each variable by including all covariates in one model.

### Patient and public involvement

Patients and/or the public were not involved in the design, conduct, reporting, or dissemination plans of this research.

### Statistical analyses

The mean (standard deviation [SD]) and percentage (number) were used to describe the continuous and categorical variables, respectively. ANOVA and ordinal *χ*^2^ test (Kruskal–Wallis test) were used to test if there was a trend of the mean of each continuous and categorical variable across a number of chronic pain groups. Cox proportional hazards regression models were used to examine the associations of a number of chronic pain sites with time to incident all-cause dementia, AD, vascular dementia, and frontotemporal dementia, where free of chronic pain served as the reference group. The follow-up period was calculated from the date of attending the baseline assessment until the date of the first incident dementia diagnosis, date of death, or censoring date (5 July 2022), whichever came first. In addition to univariable analysis, (1) analyses were also adjusted for sociodemographic, BMI, and lifestyle factors for model 1; (2) model 2—model 1 and health factors (i.e. presence of any comorbidities, use of NSAIDs, and use of opioid); (3) model 3—model 2 and psychological problems and sleep duration. Variance inflation factor (VIF) was used to determine the occurrence of multicollinearity. A variable whose VIF value is greater than 10 may merit further investigation. The proportional hazard assumptions of all analyses were validated using Schoenfeld residuals except for all-cause dementia and AD where the analyses were restricted to participants with a follow-up period of 6 years and over to meet the assumption. The proportional hazard assumption was also graphically assessed using Schoenfeld residuals plots (Additional file 1: Fig. S2). Subgroup analyses by the follow-up length (≤ 5 and > 5 years) were performed to examine the possibility that pain is a prodromal symptom of dementia thereby avoiding a possible reverse causality. Additional analyses were conducted among participants who underwent a fluid intelligence test, which measures various aspects of cognitive performance (Additional file 1: Supplementary Text 4). We detected a significant interaction between the number of chronic pain sites and age; therefore, subgroup analyses by age category (< 65 and ≥ 65 years) were performed to investigate the associations between the number of chronic pain sites and incident dementia. Competing risk analyses were performed with death and loss to follow-up as the competing causes using the Fine–Gray proportional subhazards model [35]. We also performed a sensitivity analysis using propensity score weighting based on generalised boosted models (GBM) for propensity score estimations in the context of multiple treatments [36]. All analyses were performed with the STATA software, version 17 (Stata Corp, College Station, TX 77,845, USA). A two-tailed *P* value less than 0.05 was considered statistically significant. To account for the potential issue of multiple testing, the Bonferroni correction was applied with a *P* value of 0.0125 (0.05 divided by 4 outcomes) considered significant.

## Results

**Fig. 1:**
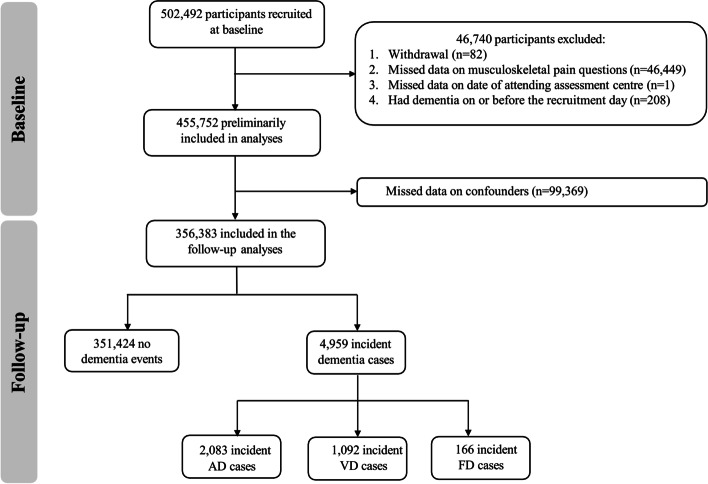
Flow chart of the study. AD, Alzheimer’s disease; VD, vascular dementia; FD, frontotemporal dementia

A total of 356,383 participants who did not have all-cause dementia at baseline and had complete data on pain and covariates were included in the observational analyses (Fig. 1). At baseline, their mean age was 56.5 (SD 8.1) years; 51.6% of participants were females. During a median follow-up of 13.3 years (interquartile range [IQR] 12.6–14.0), there were 4959 new dementia events recorded including 2083 AD, 1092 vascular dementia, and 166 frontotemporal dementia cases. The baseline characteristics of participants by number of chronic pain sites are shown in Table 1. At baseline, 58.8% of participants reported no chronic pain and 24.4%, 10.3%, 3.9%, 1.3%, and 1.3% had chronic pain in 1, 2, 3, and 4 sites and ‘all over the body’, respectively. Participants reporting a greater number of chronic pain sites appeared to have a higher proportion of developing incident all-cause dementia, AD, and vascular dementia than those without chronic pain. Compared with participants without chronic pain, participants who experienced a greater number of chronic pain sites were older, more likely to be female, a current smoker, physically inactive, and non-White ethnicity; had a greater BMI, lower education level and household income, less regular alcohol consumption, more comorbidities, higher reported use of NSAIDs and opioids; more likely to have psychological problems; and less likely to have recommended sleep duration (Table 1).

**Table 1:** Characteristics of participants at baseline, by number of chronic pain sites

Characteristics	Total	Number of chronic pain sites						
		0	1	2	3	4	Pain all over the body	*P-value
Number of participants	356,383	209,604	87,095	36,724	13,714	4666	4580	
Dementia events, % (*n*)	1.4 (4959)	1.2 (2471)	1.4 (1229)	1.9 (683)	2.2 (306)	3.0 (140)	2.8 (130)	< 0.001
Alzheimer’s disease, % (*n*)	0.6 (2083)	0.5 (1053)	0.6 (519)	0.8 (298)	0.9 (123)	1.1 (50)	0.9 (40)	< 0.001
Vascular dementia, % (*n*)	0.3 (1092)	0.3 (524)	0.3 (298)	0.4 (137)	0.4 (55)	0.7 (32)	1.0 (46)	< 0.001
Frontotemporal dementia, % (*n*)	0.05 (166)	0.04 (94)	0.05 (40)	0.05 (19)	0.04 (5)	0.09 (41)	0.09 (4)	0.575
Age (years), mean (SD)	56.5 (8.1)	56.2 (8.1)	56.6 (8.1)	57.2 (7.9)	57.8 (7.6)	58.2 (7.3)	57.0 (7.5)	< 0.001
Male, % (*n*)	48.4 (172,589)	50.2 (105,260)	47.9 (41,709)	44.5 (16,325)	42.0 (5764)	38.7 (1804)	37.7 (1727)	< 0.001
Body mass index (kg/m^2^), mean (SD)	27.4 (4.7)	26.8 (4.3)	27.7 (4.8)	28.4 (5.1)	29.3 (5.5)	30.3 (5.9)	29.8 (6.0)	< 0.001
Highest education qualification, % (*n*)								< 0.001
College or university degree/NVQ, HND, HNC, or equivalent/other professional qualifications	62.7 (223,404)	65.5 (137,354)	61.0 (53,126)	57.5 (21,125)	53.7 (7359)	50.1 (2339)	45.9 (2101)	
A levels, AS levels, or equivalent	5.8 (20,547)	6.0 (12,567)	5.7 (4950)	5.3 (1945)	4.8 (664)	4.2 (197)	4.9 (224)	
O levels, GCSEs, CSE, or equivalent	16.6 (59,159)	16.2 (33,907)	17.1 (14,903)	17.2 (6298)	17.7 (2428)	17.1 (796)	18.1 (827)	
None of the above	15.0 (53,273)	12.3 (25,776)	16.2 (14,116)	20.0 (7356)	23.8 (3263)	28.6 (1334)	31.2 (1428)	
Household income (£) before tax, % (*n*)								< 0.001
Less than 18,000	19.0 (67,646)	15.9 (33,372)	19.7 (17,119)	24.8 (9123)	31.2 (4283)	40.4 (1887)	40.7 (1862)	
18,000–30,999	22.4 (79,837)	22.1 (46,304)	22.9 (19,910)	23.1 (8465)	23.4 (3205)	21.6 (1008)	20.6 (945)	
31,000–51,999	23.6 (84,071)	24.8 (51,917)	23.3 (20,326)	21.1 (7735)	19.4 (2661)	15.4 (720)	15.6 (712)	
52,000–100,000	19.0 (67,756)	21.0 (44,017)	18.3 (15,919)	15.3 (5635)	11.0 (1505)	7.1 (332)	7.4 (339)	
Greater than 100,000	5.3 (18,726)	6.1 (12,872)	4.8 (4141)	3.5 (1281)	2.2 (302)	1.7 (80)	1.1 (50)	
Do not know/prefer not to answer	10.8 (38,356)	10.1 (21,122)	11.1 (9680)	12.2 (4485)	12.8 (1758)	13.7 (639)	14.7 (672)	
White ethnicity, % (*n*)	95.2 (339,141)	95.5 (200,220)	95.1 (82,837)	94.6 (34,729)	94.0 (12,891)	93.4 (4359)	89.6 (4105)	< 0.001
Smoking status, % (*n*)								< 0.001
Never	54.3 (193,666)	57.0 (119,379)	52.6 (45,768)	49.2 (18,083)	45.9 (6291)	42.6 (1986)	47.1 (2159)	
Former	35.4 (126,261)	34.0 (71,171)	36.6 (31,852)	38.7 (14,205)	40.5 (5547)	40.0 (1866)	35.4 (1620)	
Current	10.2 (36,456)	9.1 (19,054)	10.9 (9475)	12.1 (4436)	13.7 (1876)	17.5 (814)	17.5 (801)	
Alcohol consumption, % (*n*)								< 0.001
Daily or almost daily	21.8 (77,670)	22.8 (47,756)	21.5 (18,753)	20.2 (7428)	18.5 (2534)	14.0 (652)	11.9 (547)	
3–4 times/week	24.2 (861,220)	25.6 (53,650)	23.8 (20,739)	21.4 (7854)	18.5 (2533)	16.0 (748)	13.1 (598)	
1–2 times/week	25.7 (91,431)	26.1 (54,653)	25.7 (22,397)	24.9 (9153)	23.4 (3212)	22.4 (1043)	21.2 (973)	
1–3 times/month	10.8 (38,309)	10.3 (21,645)	11.0 (9572)	11.6 (4251)	12.3 (1684)	12.3 (575)	12.7 (582)	
Special occasions only	10.4 (37,203)	9.2 (19,176)	10.7 (9312)	12.9 (4736)	15.6 (2144)	19.2 (894)	20.5 (941)	
Never	7.2 (25,648)	6.1 (12,724)	7.3 (6322)	9.0 (3302)	11.7 (1607)	16.2 (754)	20.5 (939)	
Meeting moderate/vigorous PA recommendation, % (*n*)	54.6 (194,652)	55.9 (117,064)	54.3 (47,306)	52.7 (19,334)	50.3 (6,902)	45.8 (2139)	41.6 (1907)	< 0.001
Presence of comorbidity^a^ (≥ 1), % (*n*)	45.7 (162,767)	41.9 (87,833)	47.2 (41,065)	53.0 (19,452)	60.2 (8259)	67.1 (3129)	66.1 (3029)	< 0.001
NSAIDs use, % (*n*)	27.2 (96,906)	20.4 (42,755)	32.9 (28,661)	40.2 (14,753)	45.8 (6279)	50.0 (2331)	46.4 (2127)	< 0.001
Opioid use, % (*n*)	5.3 (18,820)	1.2 (2569)	6.1 (5332)	12.4 (4538)	22.6 (3098)	35.0 (1635)	36.0 (1648)	< 0.001
Psychological problems, % (*n*)	33.8 (120,375)	28.7 (60,050)	36.7 (31,990)	43.9 (16,110)	50.3 (6900)	56.9 (2656)	58.3 (2669)	< 0.001
Sleep duration								< 0.001
Meet the recommendation (≥ 7 and ≤ 8 h)	68.4 (243,903)	72.2 (151,374)	66.6 (58,032)	61.5 (22,573)	55.8 (7658)	48.4 (2260)	43.8 (2006)	
Less than recommendation (< 7 h)	24.1 (85,740)	21.0 (44,029)	25.6 (22,333)	30.0 (11,010)	34.2 (4688)	39.7 (1850)	40.0 (1830)	
More than recommendation (> 8 h)	7.5 (26,740)	6.8 (14,201)	7.7 (6730)	8.6 (141)	10.0 (1368)	11.9 (556)	16.2 (744)	

*Abbreviations*: *SD* Standard deviation, *PA* Physical activity, *NSAIDs* Non-steroidal anti-inflammatory drugs

^a^Self-reported any hypertension, diabetes, stroke, cardiovascular disease, cancer, or lung disease

^*^P-value was determined by ANOVA and ordinal *χ*^2^ test (Kruskal–Wallis test) (where appropriate)

**Fig. 2:**
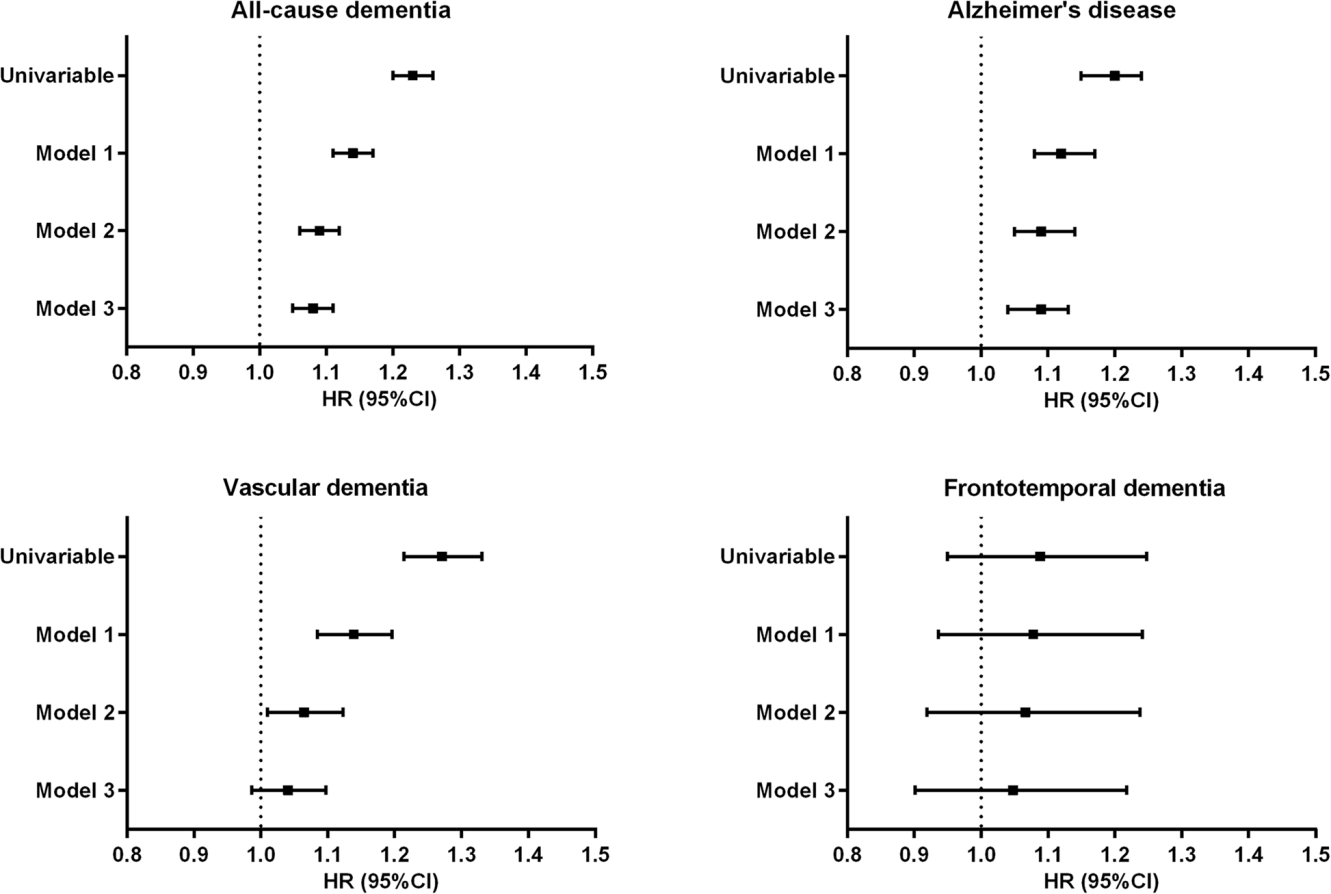
Univariable and multivariable estimates for the association between number of chronic pain sites and risk of incident dementia and its subtypes. Hazard ratio (HR) and 95% confidence interval (CI) were derived from Cox regression and refer to HR of risk of incident dementia and its subtypes associated with per one increment of the number of chronic pain sites. Model 1 adjusted for baseline age, sex, body mass index, ethnicity, highest education level, house income, smoking status, alcohol frequency, and physical activity. Model 2 further adjusted for the presence of comorbidity, non-steroidal anti-inflammatory medication use, and opioid medication use from model 1. Model 3 further adjusted for psychological problems and sleep duration from model 2

**Fig. 3:**
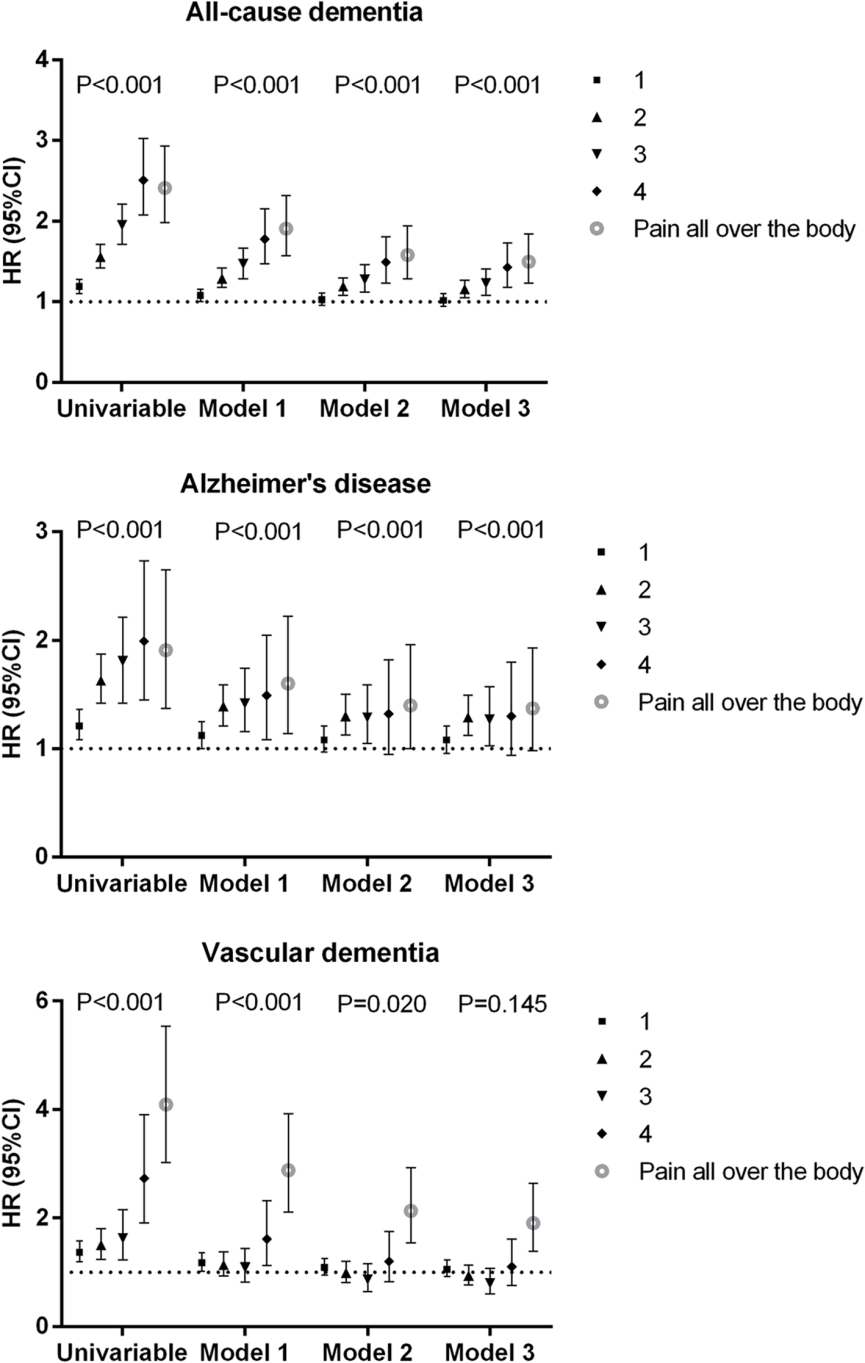
Association of number of chronic pain sites with risk of incident dementia, Alzheimer’s disease, and vascular dementia. HR (95% CI): hazard ratio (95% confidence interval) representing the HR of risk of incident dementia and its subtypes with free of chronic pain as a reference group. *P* refers to *P* for trend which was derived from Cox regression. Model 1 adjusted for baseline age, sex, body mass index, ethnicity, highest education level, house income, smoking status, alcohol frequency, and physical activity. Model 2 further adjusted for the presence of comorbidity, non-steroidal anti-inflammatory medication use, and opioid medication use from model 1. Model 3 further adjusted for psychological problems and sleep duration from model 2

The associations between the number of chronic pain sites and the risk of incident all-cause dementia and its subtypes are shown in Figs. 2 and 3 and Table 2. There was a significant association between the number of chronic pain sites and an increased risk of incident all-cause dementia in univariable analysis and even fully adjusted model (hazard ratio [HR] = 1.08 per 1 site increase, 95% confidence interval [CI] 1.05–1.11) (Fig. 2). A dose–response relationship was observed for this association with participants reporting one or more chronic pain sites showing a gradually increased risk for incident all-cause dementia, as compared to those without chronic pain (Fig. 3 and Table 2). Although the effect sizes became smaller after adjustment for covariates including sociodemographic, BMI, lifestyle, comorbidities, pain medications, psychological problems, and sleep factors, the associations remained statistically significant with participants reporting chronic pain ‘all over the body’ having the highest risk (Fig. 3 and Table 2).

**Table 2:** Associations between the number of chronic pain sites and the incidence of dementia and its subtypes over follow-up (*n* = 356,383)

	No. of participants	No. of cases (%)	No. of painful sites	Univariable model, HR (95% CI)	Model 1^a^, HR (95% CI)	Model 2^b^, HR (95% CI)	Model 3^c^, HR (95% CI)
**Dementia events**^d^	205,228	2142 (1.0)	0	Ref	Ref	Ref	Ref
	85,143	1054 (1.2)	1	**1.19 (1.10, 1.28)**^¶^	**1.08 (1.00, 1.16)**	1.03 (0.96, 1.11)	1.02 (0.95, 1.10)
	35,696	578 (1.6)	2	**1.56 (1.42, 1.71)**^¶^	**1.29 (1.18, 1.42)**^¶^	**1.19 (1.08, 1.30)**^¶^	**1.16 (1.05, 1.27)**^¶^
	13,326	267 (2.0)	3	**1.95 (1.71, 2.21)**^**¶**^	**1.47 (1.29, 1.67)**^¶^	**1.28 (1.12, 1.46)**^¶^	**1.23 (1.08, 1.41)**^¶^
	4514	116 (2.6)	4	**2.51 (2.08, 3.03)**^¶^	**1.78 (1.47, 2.15)**^¶^	**1.49 (1.23, 1.81)**^¶^	**1.43 (1.18, 1.73)**^¶^
	4360	107 (2.5)	Pain all over the body	**2.41 (1.98, 2.93)**^¶^	**1.91 (1.57, 2.32)**^¶^	**1.58 (1.29, 1.94)**^¶^	**1.50 (1.23, 1.84)**^¶^
			*P* for trend^‖^	** < 0.001**^¶^	** < 0.001**^¶^	** < 0.001**^¶^	** < 0.001**^¶^
Alzheimer’s disease^d^	205,368	929 (0.5)	0	Ref	Ref	Ref	Ref
	85,226	467 (0.6)	1	**1.21 (1.08, 1.36)**^¶^	**1.12 (1.00, 1.25)**	1.08 (0.97, 1.21)	1.08 (0.96, 1.21)
	35,744	263 (0.7)	2	**1.63 (1.42, 1.87)**^¶^	**1.39 (1.21, 1.59)**^¶^	**1.30 (1.13, 1.50)**^¶^	**1.29 (1.12, 1.49)**^¶^
	13,342	108 (0.8)	3	**1.81 (1.48, 2.21)**^¶^	**1.42 (1.16, 1.74)**^¶^	**1.29 (1.05, 1.59)**	**1.27 (1.03, 1.57)**
	4524	40 (0.9)	4	**1.99 (1.45, 2.73)**^¶^	**1.49 (1.08, 2.05)**	1.32 (0.95, 1.82)	1.30 (0.94, 1.80)
	4376	37 (0.9)	Pain all over the body	**1.91 (1.37, 2.65)**^¶^	**1.60 (1.14, 2.22)**^¶^	**1.40 (1.00, 1.96)**	1.37 (0.98, 1.93)
			*P* for trend^‖^	** < 0.001**^¶^	** < 0.001**^¶^	** < 0.001**^¶^	** < 0.001**^¶^
Vascular dementia	209,604	524 (0.25)	0	Ref	Ref	Ref	Ref
	87,095	298 (0.34)	1	**1.37 (1.19, 1.58)**^¶^	**1.18 (1.02, 1.36)**	1.09 (0.95, 1.26)	1.06 (0.92, 1.23)
	36,724	137 (0.37)	2	**1.50 (1.24, 1.81)**^¶^	1.14 (0.94, 1.38)	0.99 (0.81, 1.20)	0.94 (0.77, 1.14)
	13,714	55 (0.40)	3	**1.63 (1.23, 2.15)**^¶^	1.09 (0.82, 1.44)	0.87 (0.65, 1.16)	0.80 (0.60, 1.07)
	4666	32 (0.69)	4	**2.73 (1.91, 3.90)**^¶^	**1.61 (1.12, 2.32)**^¶^	1.20 (0.83, 1.75)	1.10 (0.76, 1.61)
	4580	46 (1.00)	Pain all over the body	**4.09 (3.03, 5.53)**^¶^	**2.88 (2.11, 3.92)**^¶^	**2.13 (1.55, 2.93)**^¶^	**1.91 (1.39, 2.64)**^¶^
			*P* for trend^‖^	** < 0.001**^¶^	** < 0.001**^¶^	**0.020**	0.145
Frontotemporal dementia	209,604	94 (0.04)	0	Ref	Ref	Ref	Ref
	87,095	40 (0.05)	1	1.02 (0.71, 1.48)	1.02 (0.70, 1.48)	1.02 (0.70, 1.48)	0.99 (0.68, 1.45)
	36,724	19 (0.05)	2	1.16 (0.71, 1.90)	1.13 (0.69, 1.86)	1.11 (0.67, 1.85)	1.07 (0.64, 1.77)
	13,714	5 (0.04)	3	0.82 (0.33, 2.01)	0.78 (0.32, 1.94)	0.76 (0.30, 1.90)	0.72 (0.28, 1.80)
	4666	4 (0.09)	4	1.93 (0.71, 5.24)	1.85 (0.67, 5.09)	1.75 (0.62, 4.94)	1.64 (0.58, 4.64)
	4580	4 (0.09)	Pain all over the body	2.00 (0.73, 5.43)	1.94 (0.70, 5.36)	1.83 (0.65, 5.18)	1.70 (0.60, 4.82)
			*P* for trend^‖^	0.223	0.298	0.399	0.547

HRs (95% CIs) in bold represent the statistically significant results (*P* < 0.05)

*Abbreviations*: *HR* Hazard ratio, *CI* Confidence interval, *Ref* Reference group

^a^Model 1: adjusted for baseline age, sex, body mass index, ethnicity, highest education level, house income, smoking status, alcohol frequency, and meeting recommended moderate/vigorous physical activity

^b^Model 2: model 1 + presence of comorbidity, non-steroidal anti-inflammatory medication use, and opioid medication use

^c^Model 3: model 2 + psychological problems and sleep duration

^d^Analyses were restricted to those with a follow-up of 6 years and over

^‖^*P* for trend was determined by Cox regression with number of chronic painful sites as a continuous variable

^¶^Significant association that passes the Bonferroni correction for multiple testing

There was also a tendency towards a higher risk of incident AD with an increasing number of chronic pain sites in univariable analysis (Figs. 2 and 3). The significant association persisted in the adjusted models. Relative to those without chronic pain, participants having 2, 3, and 4 pain sites and pain ‘all over the body’ had a 29%, 27%, 30%, and 37% higher risk of incident AD in the fully adjusted model, respectively, of which 4 pain sites and pain ‘all over the body’ were of borderline significance (Fig. 3 and Table 2). Although the trend of the association between the number of chronic pain sites and incident vascular dementia was non-significant in the fully adjusted model, participants who had chronic pain ‘all over the body’ had a higher risk in comparison with those without chronic pain (Figs. 2 and 3 and Table 2). No significant trend and association were observed between the number of chronic pain sites and incident frontotemporal dementia (Fig. 2 and Table 2).

**Fig. 4:**
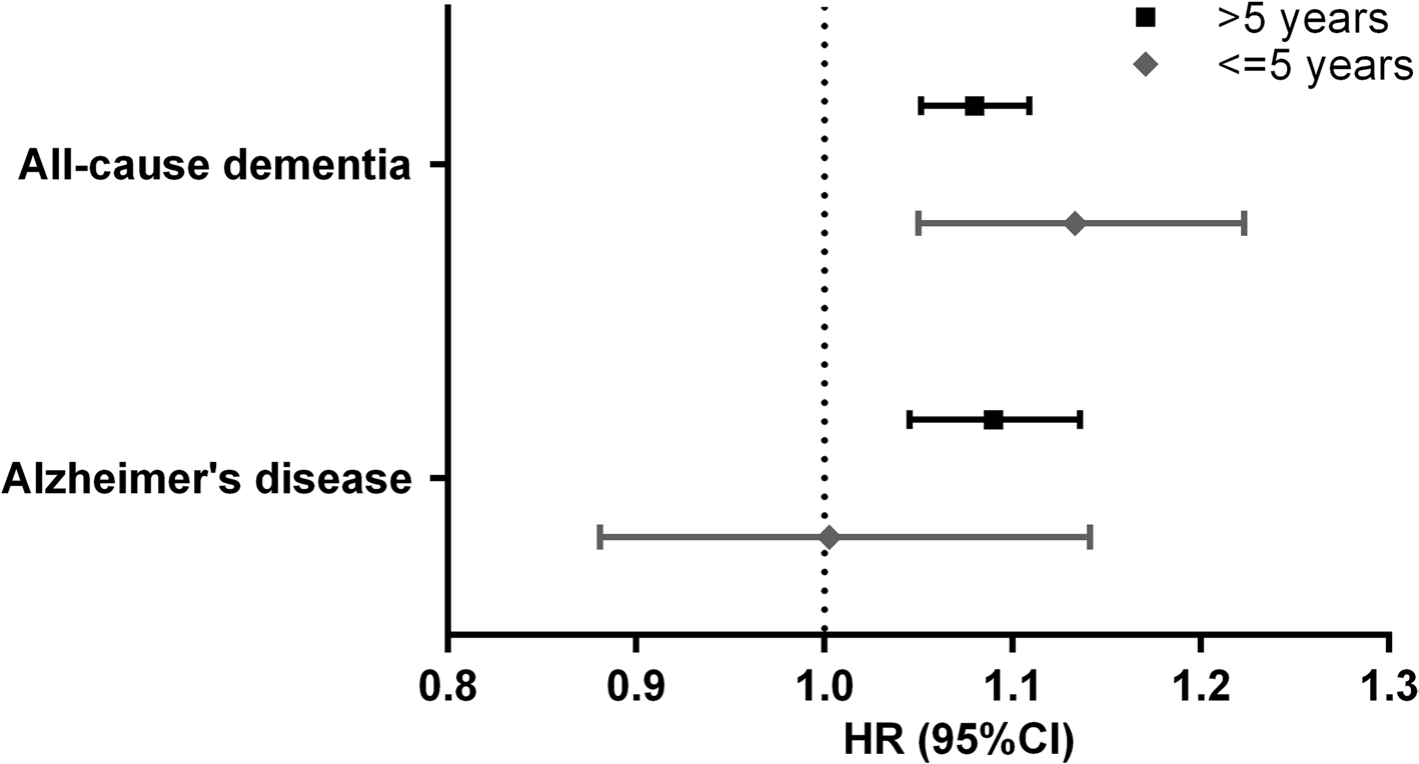
Subgroup analyses by the follow-up period on the association between number of chronic pain sites and risk of incident dementia and Alzheimer’s disease. Hazard ratio (HR) and 95% confidence interval (CI) were derived from Cox regression and refer to HR of risk of incident dementia and Alzheimer’s disease associated with per one increment of the number of chronic painful sites. Multivariable adjustment was for age, sex, body mass index, ethnicity, highest education level, house income, smoking status, alcohol frequency, physical activity, presence of comorbidity, non-steroidal anti-inflammatory medication use, opioid medication use, psychological problems, and sleep duration

**Fig. 5:**
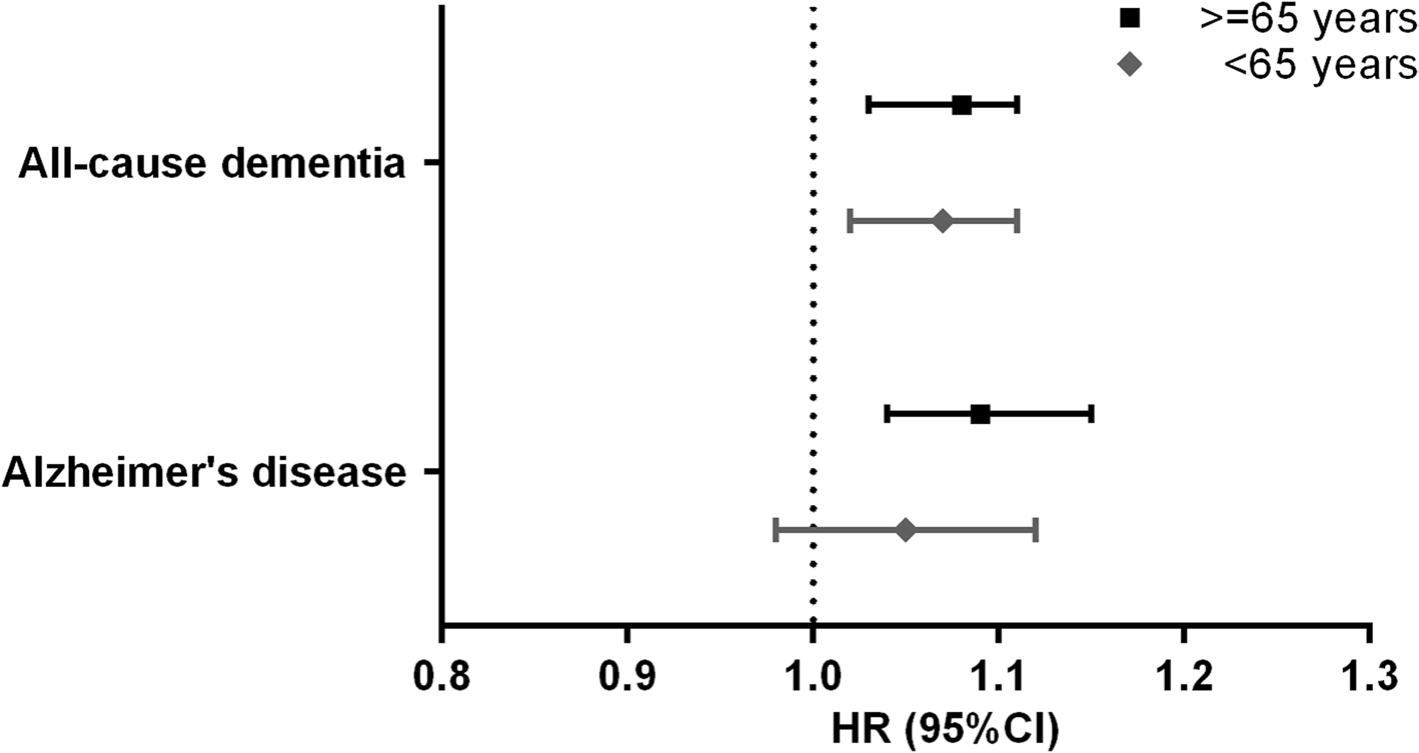
Associations of number of chronic pain sites with incident dementia and Alzheimer’s disease by age group. Hazard ratio (HR) and 95% confidence interval (CI) were derived from Cox regression and refer to HR of risk of incident dementia and Alzheimer’s disease associated with per one increment of the number of chronic painful sites. Multivariable adjustment was for age, sex, body mass index, ethnicity, highest education level, house income, smoking status, alcohol frequency, physical activity, presence of comorbidity, non-steroidal anti-inflammatory medication use, opioid medication use, psychological problems, and sleep duration

Subgroup analyses in participants with ≤ 5 and > 5 years of follow-up showed a significant association between the number of chronic pain sites and incident all-cause dementia, as shown in Fig. 4. A significant association for incident AD was only observed in those with > 5 and years of follow-up (Fig. 4). There was no significant association between the number of chronic pain sites and incident all-cause dementia among participants with the fluid intelligence test, either before or after further adjustment for fluid intelligence score (Additional file 1: Table S2). We also observed significant associations of the number of chronic pain sites with both incident all-cause dementia and AD among participants aged < 65 years and ≥ 65 years, while the effect size was slightly larger among those aged 65 years and older (Fig. 5). Competing risk analyses also revealed similar results when considering death and loss to follow-up as competing risks (Additional file 1: Table S3). In addition, after adjusting for propensity scores only and both propensity scores and other covariates, we found that the results did not substantially change, and the effect size of the associations was larger when we adjusted for propensity scores only, as shown in Additional file 1: Table S4.

## Discussion

This study leveraging data from > 356,000 individuals in a population-based cohort found that per one chronic pain site increase was associated with an 8% increased risk of incident all-cause dementia over a median follow-up of 13 years and that there was a tendency towards a higher risk of incident all-cause dementia as the number of chronic pain sites increased, with the cumulative incidence of dementia of > 40% higher in those with pain in four sites or ‘all over the body’ compared to those without chronic musculoskeletal pain. These associations remained statistically significant after controlling for potential confounding factors such as sociodemographics, BMI, lifestyle, comorbidities, pain medications, psychological problems, and sleep factors, although the effect sizes of the associations were attenuated. Similar associations were observed for AD but not for vascular and frontotemporal dementia, which may reflect the difference in mechanisms mediating different subtypes of dementia in chronic musculoskeletal pain. To the best of our knowledge, this is the first study to examine the associations between the number of chronic pain sites and the risk of all-cause dementia and its subtypes.

The findings of this study that greater number of chronic pain sites was associated with a higher risk of all-cause dementia in part agree with previous longitudinal studies showing an increased risk of dementia in individuals with chronic pain or pain conditions [18–20, 37], although these studies did not assess the number of painful sites as in the current study. Two recent retrospective US cohort studies found an increased risk of AD and related dementias in individuals with non-cancer chronic pain conditions compared to those without pain conditions during 2 years of follow-up [18, 19]. In a study with a median follow-up of 8.6 years, Whitlock et al. showed that persistent pain was associated with accelerated memory decline and increased probability of dementia [15]. Tzeng et al. also reported that fibromyalgia, a widespread musculoskeletal pain disorder, had a 2.77-fold increased risk of all-cause dementia than the age- and sex-matched control group in a study with 10 years of follow-up [37]. Similarly, analyses of the Framingham Heart Study estimated a 43% increase in all-cause dementia risk in people with widespread pain over a median follow-up of 10 years [20].

In contrast, a recent study with 24 years of follow-up in a small sample (*n* = 593) found the presence of chronic pain was not associated with all-cause dementia [28]. Another study also failed to detect a positive association between pain and cognitive decline over 4 years of follow-up [27]. Variations in the study design, study population, follow-up length, pain, and cognition measurement/dementia diagnosis between our study and the previous studies may be a part of the explanation for the discrepant results. However, previous studies seldom considered some important factors common to both pain and dementia such as sleep problems [30] and opioid use [31], thus increasing the possibility of confounding and reverse causation. The analyses of this study took into account all potential covariates, revealing that number of chronic pain sites per se is linked to an increased risk of all-cause dementia, thus providing the evidence that individuals experiencing a greater number of chronic pain sites have adverse outcomes [12–14]. Moreover, this is further strengthened by the findings from subgroup analyses that the direction and strength of the association between the number of chronic pain sites and dementia were consistent between participants ≤ 5 and > 5 years of follow-up. Of note, the significant associations between the number of chronic pain sites and all-cause dementia persisted after adjustment for covariates, but the effect sizes were slightly attenuated, suggesting the association of chronic pain with incident all-cause dementia may be partially, but not fully influenced by these covariates. Our findings appear to support a greater number of chronic pain sites as a risk factor instead of prodromal symptom for dementia [29, 38] since one would expect a positive association between number of chronic pain sites and dementia in a shorter but not a longer follow-up if chronic pain is a prodromal symptom of dementia.

In addition, the current study found that greater number of chronic pain sites was associated with incident dementia in both participants aged < 65 years and ≥ 65 years, and the effect size was larger among those aged ≥ 65 years. These findings highlight the risk of incident dementia in those with a greater number of chronic pain sites is modified by age and may reflect that the early-onset of dementia (< 65 years) may develop much earlier in life among people experiencing a greater number of chronic pain sites. Therefore, this study provides new evidence that significant associations between chronic pain and incident dementia are not only observed in older adults as previously shown [17–19, 27] but in middle-aged adults. The latter finding provides further support that the number of chronic pain sites may be a risk factor for incident dementia as the younger group is less likely to be affected by reverse causality. Although the underlying mechanisms mediating pain-dementia are not clear, it might be plausible that chronic pain may not only directly compete with cognitive processing resources and divert attention [22], but also leads to neuroinflammation and neuropathological changes involved in cognition processing and dementia [21, 23, 24].

Our analyses also provide evidence for an association between the greater number of chronic pain sites and the risk of AD, but not vascular and frontotemporal subtypes of dementia. More specifically, there is a dose–response relationship between the increasing number of chronic pain sites and incident AD after controlling for a range of potential confounders. The underlying reason for this difference is unclear as evidence on other dementia subtypes other than AD is scarce, but it may be speculated that chronic pain-induced neuroinflammation may exert a different effect on the neuropathological changes responsible for cognition processing [21, 23, 24]. However, the discrepancy could also be explained by the small number of vascular and frontotemporal dementia cases which may reduce the statistical power to detect the differences, particularly for frontotemporal dementia.

This study has several strengths. The observational analyses had a prospective design with a very large sample size and long-term follow-up, enabling us to assess the incidence of dementia. In addition, we were able to explore the relationships between pain chronicity and number of chronic pain sites and different subtypes of dementia. Detailed information on all known confounders was available, allowing us to determine whether the number of chronic pain sites is associated with the excess risk of dementia. However, several limitations of this study need to be acknowledged. First, our findings may not be generalised to other ethnicities since individuals from the UK Biobank are primarily of White British origin and there is evidence suggesting ethnic differences in pain perception [39]. In addition, participants from the UK Biobank were relatively healthy and young [40], so the overall incident rate of dementia was relatively low, leading to the overall small effects of chronic pain on dementia. Second, the relatively short follow-up periods in some individuals make it hard to rule out the possibility that pain is a prodromal symptom; however, only 1.9% of participants had ≤ 5 years of follow-up, and consistent results were found between those with ≤ 5 years and > 5 years of follow-up. Third, dementia diagnoses were from the registry-based data without detailed neuropsychological assessments, which may lead to misclassification, particularly in subtypes of dementia; however, the overall accuracy of dementia diagnoses through registries has been reported to be reasonable [33]. Fourth, although we were able to consider a range of confounding factors, residual confounding (e.g. due to baseline cognitive status) may still have occurred given the nature of the observational study. In this study, we did not find a significant association between the number of chronic pain sites and incident all-cause dementia among participants with the fluid intelligence test, either before or after further adjustment for fluid intelligence score; therefore, the significant findings observed in the whole sample may need to be interpreted with caution. One possible explanation for the lack of association is that fluid intelligence was only measured in a subsample of the cohort, and only 93,995 participants had complete data on pain, dementia, and covariates. This reduced sample resulted in a smaller number of events in each pain group, particularly in those with pain in four sites (which had only 11 cases) and ‘all over the body’ (which had only three cases). However, we found similar results when we excluded those with ≤ 5 years of follow-up (Fig. 4), which may have helped to minimise the influence of baseline cognitive status. Moreover, previous research has shown that pain is longitudinally associated with cognitive decline [15, 16], suggesting that cognitive function may play a mediating role, rather than being a confounder, in the relationship between pain and dementia. Therefore, adjusting for cognitive status in the model may have resulted in over-adjustment and loss of statistical power. Lastly, the severity of pain was not measured in the UK Biobank, so we were unable to explore the association between pain severity and incident dementia. However, evidence suggests that widespread pain or pain in multiple sites is indicative of increased pain sensitisation in the central nervous system [41–43], which is positively correlated with pain severity [44]. It may be reasonable to postulate that increasing pain severity may predispose people to develop dementia as well. Furthermore, this is an observational study, so whether participants with chronic pain may have been taking pain management strategies is unknown. We, therefore, cannot determine whether pain would last in the future.

## Conclusions

Greater number of chronic pain sites was associated with an increased risk of incident all-cause dementia and AD. The association was not fully explained by a range of potential confounders including sociodemographic, BMI, lifestyle, comorbidities, pain medications, psychological problems, and sleep factors. These findings suggest that chronic pain in multiple sites may represent an accessible marker to assess an individual’s dementia risk and identify ‘at-risk’ individuals at early stages.

## Supplementary Information

**Additional file 1: Supplementary Text 1. **Criteria for defining non-steroidal anti-inflammatory drugsuse. **Supplementary Text 2.** Criteria for defining opioid use. **Supplementary Text 3.** Detailed description of defining psychological problem and sleep duration. **Supplementary Text 4.** Fluid intelligence test. **Table S1.** International Classification of Disease codes used to ascertain dementia and its subtypes. **Table S2.** Associations between number of chronic pain sites and incident dementia among those with fluid intelligence test. **Table S3.** Competing risk analyses of the associations between number of chronic pain sites and incidence of dementia and its subtypes. **Table S4.** Associations between number of chronic pain sites and incidence of dementia and its subtypes using propensity score weighting. **Fig. S1.** The presumed relationships between the selected covariate subsets and pain/dementia. **Fig. S2.** Scaled Schoenfeld residuals plots to assess the proportional hazard assumption. Scaled Schoenfeld residuals were from Cox models with number of chronic pain sites as the predictor, adjusting for age, sex, body mass index, ethnicity, highest education level, house income, smoking status, alcohol frequency, physical activity, presence of comorbidity, non-steroidal anti-inflammatory medication use, opioid medication use, psychological problems, and sleep duration. The *p*-value was from the Schoenfeld test on the corresponding Cox model, with a *P* >0.05 indicating no violation of the assumption.

## Data Availability

The datasets used and/or analysed during the current study are available from the corresponding author upon reasonable request.

## Abbreviations

AD: Alzheimer’s disease
BMI: Body mass index
CI: Confidence interval
HR: Hazard ratio
ICD: International Classification of Diseases
NCPS: Number of chronic pain sites
NHS: National Health Service
NSAIDs: Non-steroidal anti-inflammatory drugs
PA: Physical activity
SD: Standard deviation
VIF: Variance inflation factor
