# Supplementary material for: Association between chronic pain and risk of incident dementia: findings from a prospective cohort
Source: BMC Med. 2023 May 4;21:169. doi: 10.1186/s12916-023-02875-x (PMC10161483; doi:10.1186/s12916-023-02875-x)
Supplement: Supplementary file 1 — Additional file 1: Supplementary Text 1. Criteria for defining non-steroidal anti-inflammatory drugsuse. Supplementary Text 2. Criteria for defining opioid use. Supplementary Text 3. Detailed description of defining psychological problem and sleep duration. Supplementary Text 4. Fluid intelligence test. Table S1. International Classification of Disease codes used to ascertain dementia and its subtypes. Table S2. Associations between number of chronic pain sites and incident dementia among those with fluid intelligence test. Table S3. Competing risk analyses of the associations between number of chronic pain sites and incidence of dementia and its subtypes. Table S4. Associations between number of chronic pain sites and incidence of dementia and its subtypes using propensity score weighting. Fig. S1. The presumed relationships between the selected covariate subsets and pain/dementia. Fig. S2. Scaled Schoenfeld residuals plots to assess the proportional hazard assumption. Scaled Schoenfeld residuals were from Cox models with number of chronic pain sites as the predictor, adjusting for age, sex, body mass index, ethnicity, highest education level, house income, smoking status, alcohol frequency, physical activity, presence of comorbidity, non-steroidal anti-inflammatory medication use, opioid medication use, psychological problems, and sleep duration. The p-value was from the Schoenfeld test on the corresponding Cox model, with a P >0.05 indicating no violation of the assumption. [file 12916_2023_2875_MOESM1_ESM.docx]

**Additional file 1**

**SUPPLEMENTARY TEXT – 1**

**Criteria for defining non-steroidal anti drugs (NSAIDs) use**

The use of NSAIDs was defined if participants reported currently taking any of the following medications during the verbal interview at baseline [ID 20003]. NSAIDs codes [UK Biobank Data-coding 4]: 1140861806, 1140864860, 1140868226, 1140868282, 1140872040, 1140882108, 1140882190, 1140882268, 1140882392, 1141163138, 1141164044, 1141167844, 1140909772, 1140925942, 1140861808, 1140882192, 1141176662, 1141176668, 1141176670, 1140884488, 1140878036, 1140877868, 1140877872, 1140877874, 1140871168, 1140871174, 1140877892, 1140878034, 1140921828, 1141176878, 1141191028, 1141182674, 1140871282, 1140871284, 1140871188, 1140871196, 1141193170, 1140871310, 1140878030, 1140910496, 1140911748, 1140911750, 1141153134, 1141157412, 1141184546, 1141194296, 1141200748, 1140871406, 1140928656, 1141153082, 1140911754, 1141153134, 1141188516, 1140871374, 1141191742, 1140871388, 1141149110, 1141190952, 1141182868, 1140853090, 1141181752, 1141164874, 1141178894, 1141187776, 1140871336, 1141157452, 1140909936, 1141181656, 1140871506, 1140875642, 1141164746, 1140871516, 1140871522, 1140877894, 1140871528, 1140871532, 1140884558, 1140916866, 1140917406, 1140927756, 1140851142, 1140884558, 1140881118, 1140875336, 1140875338, 1140871462, 1140881612, 1140871472, 1140871462, 1140881612, 1140871666, 1140875546, 1141169526, 1141182754, 1140871672, 1140877954, 1140871092, 1140871094, 1140871604, 1140871606, 1140875268, 1140875270, 1140853030.

**SUPPLEMENTARY TEXT – 2**

**Criteria for defining opioid use**

Participants were defined using opioid if they self-reported currently taking any of the following medications during the verbal interview at baseline [ID 20003]. Opioid codes: 1140864992, 1141190956, 1140884444, 1140884464, 1140856406, 1140878030, 1140882268, 1140882392, 1140882394, 1140882396, 1140865654, 1140884464, 1140871692, 1140882114, 1140882116, 1140882406, 1140884460, 1140910376, 1140910402, 1141171038, 1140879212, 1140880956, 1141157470, 1140884388, 1140884482, 1140922628, 1140888836, 1140871732, 1140923346, 1140923348, 1140923350, 1140923344.

**SUPPLEMENTARY TEXT – 3**

**Detailed description of defining psychological problem and sleep duration**

Psychological problem was measured using the following two questions at baseline: ‘Have you ever seen a general practitioner for nerves, anxiety, tension or depression?’, and ‘Have you ever seen a psychiatrist for nerves, anxiety, tension or depression?’. Participants were classified as having psychological problem if they answered ‘yes’ to either of them. Sleep duration was self-reported using a standard question: ‘About how many hours sleep do you get in every 24 h? including naps’. Responses with hourly increments were categorised into: less than recommendation (<7 hours), meet the recommendation (≥7 and ≤8 hours) and more than recommendation (>8 hours).

**SUPPLEMENTARY TEXT – 4**

**Fluid intelligence test**

Fluid intelligence test, also known as the verbal-numerical reasoning test, was introduced part-way through the baseline assessment period in the UK Biobank study. Participants were required to solve as many multiple choice questions as possible (maximum 13) within 2 minutes. The test comprised six verbal items and seven numerical items, involving sequence recognition and arithmetic. Any incorrect or unattempted questions were scored as zero. The total number of correct answers (maximum 13) was calculated for the analysis.

**Table S1** International Classification of Disease codes used to ascertain dementia and its subtypes

|  | **ICD-9** | **ICD-10** |
| --- | --- | --- |
| Dementia | 290.2, 290.3, 290.4, 291.2, 294.1, 331.0, 331.1, 331.2, 331.5 | A81.0, F00, F00.0, F00.1, F00.2, F00.9, F01, F01.0, F01.1, F01.2, F01.3, F01.8, F01.9, F02, F02.0, F02.1, F02.2, F02.3, F02.4, F02.8, F03, F05.1, F10.6, G30, G30.0, G30.1, G30.8, G30.9, G31.0, G31.1, G31.8, I67.3 |
|  |  |  |
| Alzheimer’s disease | 331.0 | F00, F00.0, F00.1, F00.2, F00.9, G30, G30.0, G30.1, G30.8, G30.9 |
|  |  |  |
| Vascular dementia | 290.4 | F01, F01.0, F01.1, F01.2, F01.3, F01.8, F01.9, I67.3 |
|  |  |  |
| Frontotemporal dementia | 331.1 | F02.0, G31.0 |

Abbreviations: ICD, International Classification of Disease

**Table S2 Associations between number of chronic pain sites and incident dementia among those with fluid intelligence test**

|  | No. of participants | No. of cases (%) | No. of painful sites | Model 1^*^ |  | Model 2^†^ |
| --- | --- | --- | --- | --- | --- | --- |
|  |  |  |  | HR (95% CI) |  | HR (95% CI) |
| **Dementia** |  |  |  |  |  |  |
|  | 59,113 | 272 (0.46) | 0 | Ref |  | Ref |
|  | 21,992 | 103 (0.47) | 1 | 0.92 (0.73, 1.16) |  | 0.90 (0.72, 1.13) |
|  | 8,507 | 58 (0.68) | 2 | 1.17 (0.87, 1.57) |  | 1.12 (0.83, 1.50) |
|  | 2,840 | 23 (0.81) | 3 | 1.14 (0.73, 1.78) |  | 1.08 (0.69, 1.68) |
|  | 816 | 11 (1.35) | 4 | 1.85 (0.99, 3.46) |  | 1.68 (0.90, 3.14) |
|  | 727 | 3 (0.41) | Pain all over the body | 0.58 (0.18, 1.83) |  | 0.50 (0.16, 1.58) |
|  |  |  | P for trend^‡^ | 0.375 |  | 0.699 |

Abbreviations: HR, hazard ratio; CI, confidence interval; Ref, reference group.

*Adjusted for age, sex, body mass index, ethnicity, highest education level, house income, smoking status, alcohol frequency, physical activity, presence of comorbidity, nonsteroidal anti-inflammatory medication use, opioid medication use, psychological problems, and sleep duration.

†Further adjusted for fluid intelligence.

‡P for trend was determined by Cox regression with number of chronic painful sites as a continuous variable.

**Table S3 Competing risk analyses of the associations between number of chronic pain sites and incidence of dementia and its subtypes**

|  | No. of participants | No. of cases (%) | No. of painful sites | Univariable model |  | Model 1^*^ | |  | | Model 2^†^ | |  | | Model 3^‡^ | |  |
| --- | --- | --- | --- | --- | --- | --- | --- | --- | --- | --- | --- | --- | --- | --- | --- | --- |
|  |  |  |  | HR (95% CI) |  | HR (95% CI) | |  | | HR (95% CI) | |  | | HR (95% CI) | |  |
| **Dementia events**^§^ | 205,228 | 2142 (1.0) | 0 | Ref |  | Ref | |  | | Ref | |  | | Ref | |  |
|  | 85,143 | 1,054 (1.2) | 1 | **1.19 (****1.10, 1.28)** |  | **1.08 (****1.01, 1.16)** | |  | | 1.04 (0.96, 1.12) | |  | | 1.02 (0.95, 1.10) | |  |
|  | 35,696 | 578 (1.6) | 2 | **1.55 (****1.41, 1.70)** |  | **1.29 (****1.18, 1.42)** | |  | | **1.19 (****1.08, 1.31)** | |  | | **1.16 (****1.05, 1.27)** | |  |
|  | 13,326 | 267 (2.0) | 3 | **1.92 (****1.69, 2.18)** |  | | **1.45 (****1.27, 1.65)** | |  | | **1.27 (****1.11, 1.45)** | |  | | **1.22 (****1.07, 1.40)** | |
|  | 4,514 | 116 (2.6) | 4 | **2.46 (****2.04, 2.97)** |  | | **1.77 (****1.46, 2.14)** | |  | | **1.50 (****1.24, 1.82)** | |  | | **1.44 (****1.19, 1.74)** | |
|  | 4,360 | 107 (2.5) | Pain all over the body | **2.35 (****1.94, 2.86)** |  | | **1.87 (****1.53, 2.28)** | |  | | **1.57 (****1.28, 1.92)** | |  | | **1.49 (****1.22, 1.82)** | |
|  |  |  | P for trend^‖^ | **<0.001** |  | | **<0.001** | |  | | **<0.001** | |  | | **<0.001** | |
| Alzheimer’s disease^§^ | 205,368 | 929 (0.5) | 0 | Ref |  | Ref | |  | | Ref | |  | | Ref | |  |
|  | 85,226 | 467 (0.6) | 1 | **1.21 (****1.08, 1.35)** |  | **1.12 (****1.00, 1.25)** | |  | | 1.09 (0.97, 1.22) | |  | | 1.08 (0.97, 1.21) | |  |
|  | 35,744 | 263 (0.7) | 2 | **1.62 (****1.42, 1.86)** |  | **1.38 (****1.20, 1.59)** | |  | | **1.30 (****1.13, 1.50)** | |  | | **1.29 (****1.12, 1.49)** | |  |
|  | 13,342 | 108 (0.8) | 3 | **1.78 (****1.46, 2.18)** |  | **1.****40 (1.15, 1.71)** | |  | | **1.28 (****1.04, 1.57)** | |  | | **1.27 (****1.03, 1.55)** | |  |
|  | 4,524 | 40 (0.9) | 4 | **1.95 (****1.42, 2.67)** |  | **1.49 (****1.08, 2.05)** | |  | | 1.33 (0.96, 1.83) | |  | | 1.31 (0.95, 1.81) | |  |
|  | 4,376 | 37 (0.9) | Pain all over the body | **1.86 (****1.34, 2.58)** |  | **1.56 (****1.12, 2.17)** | |  | | 1.38 (0.99, 1.93) | |  | | 1.36 (0.97, 1.90) | |  |
|  |  |  | P for trend^‖^ | **<0.001** |  | **<0.001** | |  | | **<0.001** | |  | | **<0.001** | |  |
| Vascular dementia | 209,604 | 524 (0.25) | 0 | Ref |  | Ref | |  | | Ref | |  | | Ref | |  |
|  | 87,095 | 298 (0.34) | 1 | **1.37 (****1.19, 1.58)** |  | **1.18 (****1.03, 1.37)** | |  | | 1.11 (0.96, 1.28) | |  | | 1.07 (0.93, 1.24) | |  |
|  | 36,724 | 137 (0.37) | 2 | **1.49 (****1.23, 1.80)** |  | 1.14 (0.94, 1.39) | |  | | 0.99 (0.81, 1.21) | |  | | 0.94 (0.77, 1.14) | |  |
|  | 13,714 | 55 (0.40) | 3 | **1.60 (****1.21, 2.11)** |  | 1.08 (0.81, 1.43) | |  | | 0.86 (0.65, 1.16) | |  | | 0.80 (0.60, 1.07) | |  |
|  | 4,666 | 32 (0.69) | 4 | **2.73 (****1.91, 3.90)** |  | **1.69 (****1.18, 2.44)** | |  | | 1.28 (0.89, 1.85) | |  | | 1.17 (0.81, 1.70) | |  |
|  | 4,580 | 46 (1.00) | Pain all over the body | **4.01 (****2.97, 5.42)** |  | **2.75 (****2.02, 3.75)** | |  | | **2.06 (****1.49, 2.84)** | |  | | **1.85 (****1.34, 2.56)** | |  |
|  |  |  | P for trend^‖^ | **<0.001** |  | **<0.001** | |  | | **0.021** | |  | | 0.146 | |  |

HRs (95% CIs) in bold represent statistically significant results. Abbreviations: HR, hazard ratio; CI, confidence interval; Ref, reference group.

*Model 1: adjusted for baseline age, sex, body mass index, ethnicity, highest education level, house income, smoking status, alcohol frequency, meeting recommended moderate/vigorous physical activity. †Model 2: Model 1 + presence of comorbidity, nonsteroidal anti-inflammatory medication use, and opioid medication use. ‡Model 3: Model 2 + psychological problems and sleep duration.

§Analyses were restricted to those with a follow-up of 6 years and over.

‖P for trend was determined by Cox regression with number of chronic painful sites as a continuous variable.

**Table S4 Associations between number of chronic pain sites and incidence of dementia and its subtypes using propensity score weighting**

|  | No. of painful sites | Model 1^*^ |  | Model 2^†^ |
| --- | --- | --- | --- | --- |
|  |  | HR (95% CI) |  | HR (95% CI) |
| **Dementia events**^‡^ | 0 | Ref |  | Ref |
|  | 1 | **1.21 (1.12, 1.30)** |  | 1.02 (0.95, 1.10) |
|  | 2 | **1.66 (1.51, 1.82)** |  | **1.17 (1.06, 1.29)** |
|  | 3 | **2.31 (2.01, 2.65)** |  | **1.25 (1.09, 1.45)** |
|  | 4 | **3.69 (2.99, 4.56)** |  | **1.49 (1.19, 1.87)** |
|  | Pain all over the body | **3.50 (2.82, 4.34)** |  | **1.57 (1.24, 1.99)** |
|  | P for trend^§^ | **<0.001** |  | **<0.001** |
| Alzheimer’s disease^‡^ | 0 | Ref |  | Ref |
|  | 1 | **1.25 (1.11, 1.39)** |  | 1.10 (0.98, 1.23) |
|  | 2 | **1.79 (1.55, 2.06)** |  | **1.37 (1.18, 1.59)** |
|  | 3 | **2.31 (1.85, 2.89)** |  | **1.46 (1.15, 1.84)** |
|  | 4 | **3.29 (2.31, 4.69)** |  | **1.76 (1.19, 2.61)** |
|  | Pain all over the body | **3.11 (2.17, 4.46)** |  | **1.87 (1.25, 2.81)** |
|  | P for trend^§^ | **<0.001** |  | **<0.001** |
| Vascular dementia | 0 | Ref |  | Ref |
|  | 1 | **1.42 (1.22, 1.66)** |  | 1.08 (0.92, 1.26) |
|  | 2 | **1.66 (1.35, 2.04)** |  | 0.94 (0.76, 1.17) |
|  | 3 | **2.28 (1.65, 3.12)** |  | 0.87 (0.63, 1.19) |
|  | 4 | **5.02 (3.30, 7.67)** |  | 1.26 (0.80, 1.99) |
|  | Pain all over the body | **6.77 (4.65, 9.87)** |  | **1.95 (1.28, 2.99)** |
|  | P for trend^§^ | **<0.001** |  | 0.277 |

HRs (95% CIs) in bold represent statistically significant results. Abbreviations: HR, hazard ratio; CI, confidence interval; Ref, reference group.

*Adjusted for propensity scores only.

†Adjusted for age, sex, body mass index, ethnicity, highest education level, house income, smoking status, alcohol frequency, physical activity, presence of comorbidity, nonsteroidal anti-inflammatory medication use, opioid medication use, psychological problems, sleep duration, and propensity scores.

‡Analyses were restricted to those with a follow-up of 6 years and over.

§P for trend was determined by Cox regression with number of chronic painful sites as a continuous variable.


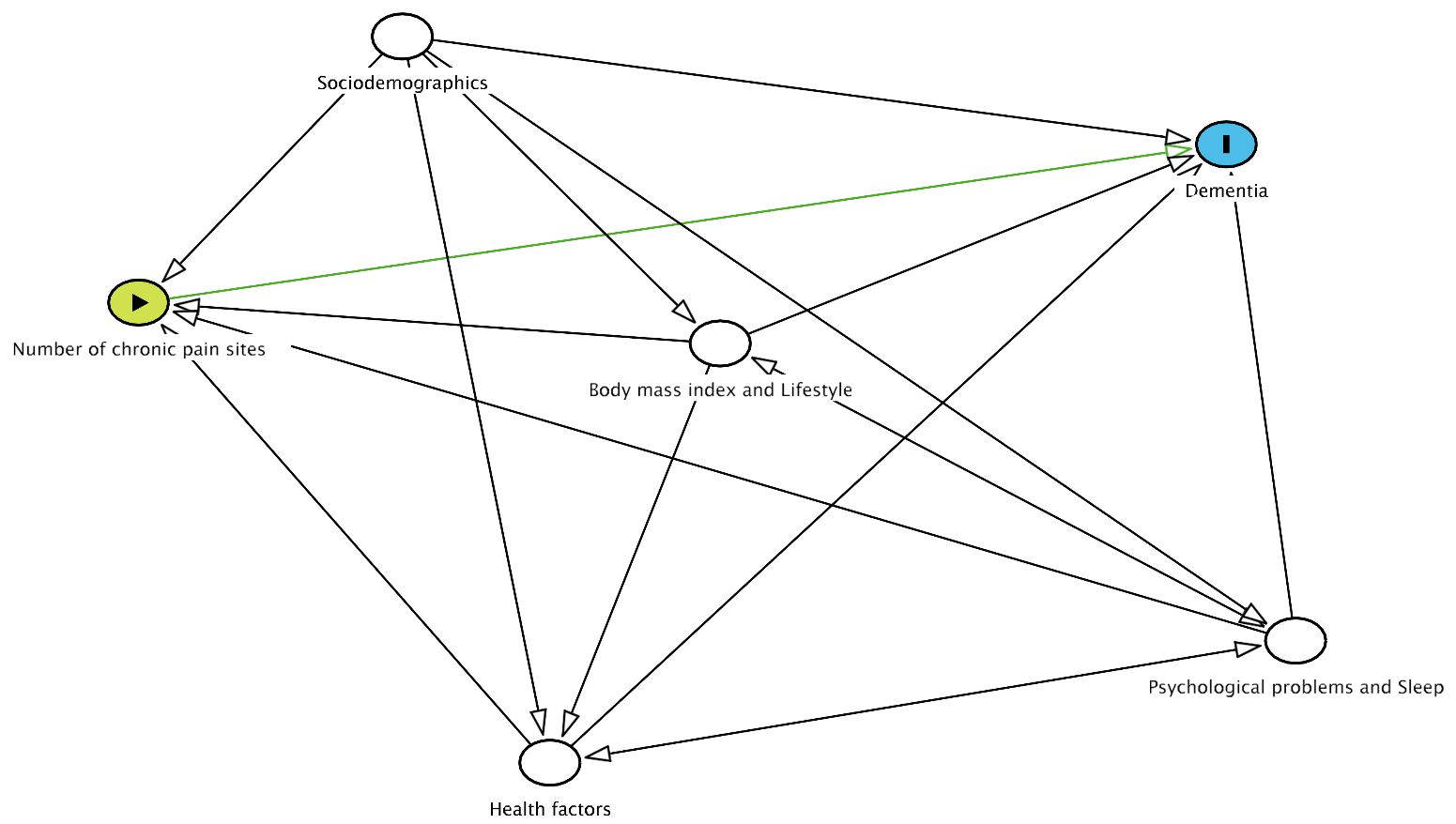
**Figure S1 The presumed relationships between the selected covariate subsets and pain/dementia.**


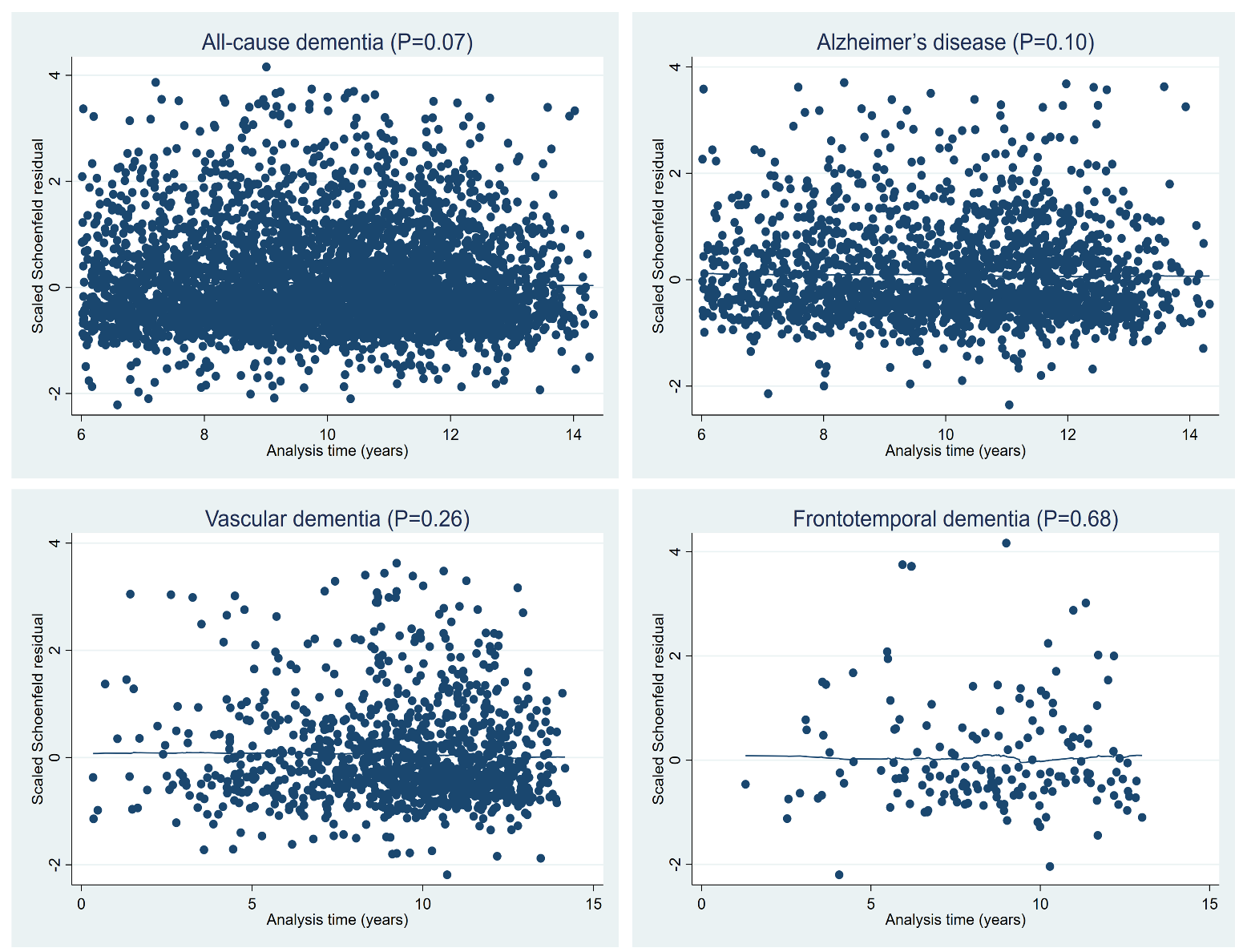
**Figure S2 Scaled Schoenfeld residuals plots to assess the proportional hazard assumption.** Scaled Schoenfeld residuals were from Cox models with number of chronic pain sites as the key predictor, adjusting for age, sex, body mass index, ethnicity, highest education level, house income, smoking status, alcohol frequency, physical activity, presence of comorbidity, nonsteroidal anti-inflammatory medication use, opioid medication use, psychological problems, and sleep duration. The p-value was from the Schoenfeld test on the corresponding Cox model, with a P >0.05 indicating no violation of the assumption.
